# Methods of Delivery in Pregnant Women with Lumbar Disc Herniation: A Narrative Review of General Management and Case Report

**DOI:** 10.25122/jml-2020-0166

**Published:** 2020

**Authors:** Francesca Gabriela Paslaru, Andrei Giovani, George Iancu, Anca Panaitescu, Gheorghe Peltecu, Radu Mircea Gorgan

**Affiliations:** 1. Neurosurgical Department, Bagdasar-Arseni Clinical Emergency Hospital, Bucharest, Romania; 2. “Carol Davila” University of Medicine and Pharmacy, Bucharest, Romania; 3. Obstetrics and Gynecology Department, Clinical Hospital of Obstetrics and Gynecology Filantropia, Bucharest, Romania

**Keywords:** Pregnancy, delivery, lumbar disc herniation

## Abstract

Low back pain is a common complaint during pregnancy, affecting approximately half of pregnant women. However, true disc herniation is extremely rare, and the majority of patients heal without surgery. The purpose of this study was to provide an overview of conservative management strategies and delivery modes for pregnant patients suffering from lumbar disc herniation without severe neurologic deficits that would require emergency surgery. We performed a narrative review of the literature using the PubMed database. Thirty-one articles were originally retrieved, out of which 7 met the inclusion criteria, compiling a total of 10 cases of parturient patients with lumbar disc hernia treated conservatively until childbirth. The preferred delivery mode was a Cesarean section, which was performed in 6 out of 10 cases. Two patients developed the cauda equina syndrome, one during a failed induction and the other patient 4 weeks after vacuum extraction. However, the second patient failed to improve after surgery. No patients developed cauda equina syndrome during or after the Cesarean section. Based on limited data, the Cesarean section seems to be preferred compared to vaginal delivery to avoid worsening symptoms and progression to the cauda equina syndrome.

## Introduction

Low back pain (LBP) during pregnancy is a very common symptom, affecting approximately half of pregnant women. Some authors suggest that the same hormonal factors determining changes in the pelvic joints could affect the intervertebral discs and posterior longitudinal ligament, promoting lumbar disk protrusion, resulting in LBP [1]. However, true disc herniation affecting pregnant women has been estimated to occur in 1 of 10,000 patients with LBP [2]. Based on available data, no more than 15% of lumbar disc hernias result in severe neurologic deficits, the cauda equina syndrome being the main reason patients require emergency surgical intervention [3]. Furthermore, the majority of patients with radiculopathy caused by lumbar disc herniation are healing without surgery, either spontaneously or with medical treatment [4]. When treating a pregnant woman, one has to simultaneously think about the health of two patients, the mother, and the unborn child. Parturient patients require a multidisciplinary team, with specialists in obstetrics, maternal-fetal medicine, neurosurgery and anesthesiology.

Since conservative management has been proven to be highly effective and remains the initial treatment of choice [4], if the patient has no neurologic deficits, the actual number of neurosurgical interventions for the herniated lumbar disc during pregnancy is very low. Most of these patients will go on to have a normal pregnancy, leading to childbirth [3].

The most suitable method of delivery for pregnant women suffering from lumbar disc herniation has not been clearly established. The purpose of the present paper is to gain further insight into this issue by performing a narrative review of the data available in the current literature.

## Material and Methods

The authors have conducted a literature search using the PubMed database for the following keywords: “pregnant” or “pregnancy”, “lumbar disc hernia” or “lumbar disc herniation” or “lumbar herniated disc” or “lumbar radiculopathy”, “childbirth” or “delivery”, combined using the terms “AND” and “OR”. A thorough hand search was also performed. The filters used were: humans, English language, adults, abstract available, full text available, academic journals.

Inclusion criteria were English language studies, studies on adult pregnant women, both case reports and reviews, abstracts, and full text available. Exclusion criteria were: articles written in languages other than English, articles regarding cases that required surgery, or in which surgery was performed postpartum. Duplicates were checked. Thirty-one articles were initially identified, but 24 were either not relevant to this topic or written in a foreign language. After applying all selection criteria, 7 articles remained and were included in the present study.

## Results

Out of the 31 articles that were initially search-retrieved, only 7 different articles met the inclusion criteria, and 10 cases were extracted [5-10]. We were not able to perform a quantitative systematic review due to the nature of the articles, being either case reports or small case series.

The mean age of the mother was 32.8 years, and the mean gestational age at diagnosis was 28 weeks. Out of 10 reported cases, 2 patients were diagnosed with lumbar disc hernia during the first trimester and 8 patients during the third trimester. 

Gestational age, signs and symptoms at diagnosis, radiographic findings are listed in Table 1. Management strategy, delivery method, and postpartum sequelae are listed in Table 2.

**Table 1:** Gestational age, signs and symptoms at diagnosis, radiographic findings.

Nr. Crt.	First author	Year	Age of the mother	Gestational age at symtoms onset	Signs and symtoms	Radiographic findings
1	Timothy J.	1999	37	33 w	Persistent numbness of the lower back with spread to perineum, urinary incontinence, LBP	L5/S1 median disc hernia
2	Al-areibi A.	2007	33	35 w	Weakness of the right lower limb muscles, loss of sensation in the S1 nerve root distribution, bowel and bladder dysfunction	L5/S1 median disc hernia
3	La Ban M.	1995	35	10 w	Acute low back pain	L5/S1 median and paramedian disc hernia compressing the left S1 nerve root
4	Jones C.S.	2015	29	38 w	Severe lower back pain and bilateral radicular leg pain at presentation, difficulty in passing urine 12h after induction of labour	L4/L5 median disc hernia
5	La Ban M.	1995	35	29 w	Mild weakness in the left tibialis anterior (TA) and exterior hallucis longus (EHL) muscles, decreased sensation of the left lateral calf and medial foot	L4/L5 dis hernia compressing the left L5 root
6			32	29 w	Low back pain, weak right extensor hallucis longus	L5/S1 dis hernia compressing the right S1 nerve root
7			30	32 w	Right buttock and lower extremity pain, sacroiliac joint tenderness, restricted right straight leg raising at 30°, and positive crossed straight leg raising at 45°	L4/L5 disc hernia compressing the left L5 root
8			35	10 w	Low back pain	L5/S1 median and paramedian disc hernia compressing the left S1 nerve root
9	Ochi H.	2014	33	32 w	Low back pain and the left-sided leg pain below the knee	L4/L5 disc paramedian disc hernia compressing the left L5 nerve root
10	Connolly T.	2019	29	32 w	Low back pain with irradiation to lower extremites	L5/S1 median disc hernia

**Table 2:** Management strategy, delivery method and postpartum sequelae.

Nr. Crt.	First author	Year	Management strategy	Gestational age at delivery	Delivery method	CES	Sequelae
1	Timothy J.	1999	Discectomy 4 weeks after delivery	38 w	Vacuum extraction	Yes	Bladder and bowel disfunction
2	Al-areibi A.	2007	Laminectomy immediately following Caesarean delivery	35 w	Caesarean delivery	Yes	No
3	La Ban M.	1995	Conservative	N/A	N/A	No	No
4	Jones C.S.	2015	Microdiscectomy after delivery	39 w	Failed labour induction followed by Caesarean section	Yes, after delivery	No
5	La Ban M.	1995	Conservative	34 w	Vaginal delivery, spontaneous preterm rupture of the membranes	No	No
6			Conservative	39 w	Caesarean section	No	No
7			Conservative	“term”	Caesarean section	No	No
8			Conservative	“term”	Caesarean section	No	No
9	Ochi H.	2014	Conservative treatment until 34 weeks, discectomy immediate following Caesarean section due to worsening of the symtoms	34 w	Caesarean section	No	No
10	Connolly T.	2019	Conservative: epidural steroid injection	N/A	Vaginal delivery	No	No

Among the 10 reviewed cases, one parturient patient presented with cauda equina syndrome (CES), gave birth by Cesarean section, underwent L5/S1 laminectomy immediately after delivery, and had no postoperative neurological deficit [7].

One patient underwent labor induction at 39 gestational weeks and proceeded for vaginal delivery, failed induction, and gave birth by Cesarean section. This patient developed CES during labor induction and underwent microdiscectomy after delivery. The radicular leg pain was resolved immediately after surgery, and her bladder control gradually recovered during the following 12 days [8]. Another patient gave birth through vacuum extraction and was readmitted 4 weeks following delivery with fecal and urinary incontinence, which failed to improve after surgery [6].

The preferred delivery method was a Cesarean section performed in 6 out of 10 cases.

## Case report

**Figure 1:**
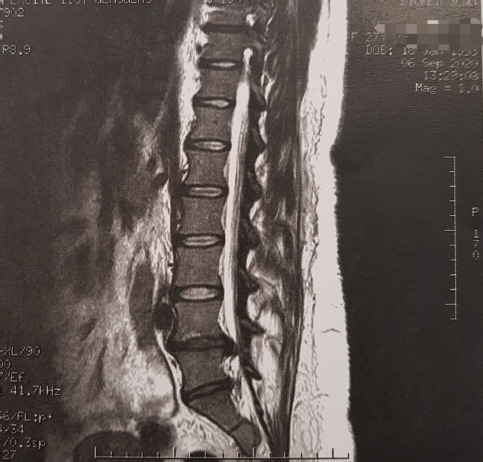
T2-weighted MRI scan, sagittal section, showing a large L4/L5 lumbar disc herniation with a caudally-migrated disc fragment.

**Figure 2:**
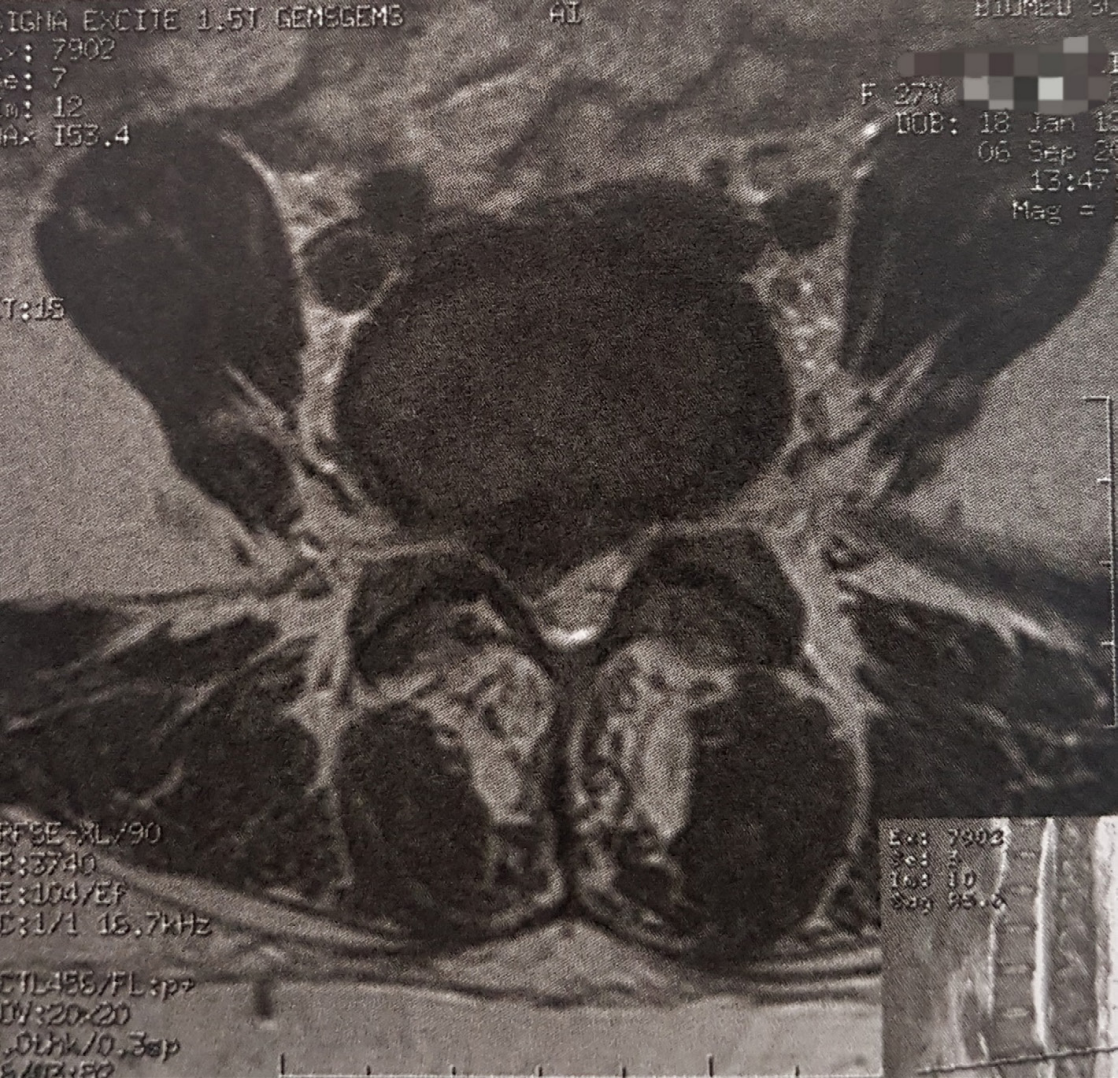
T2-weighted MRI scan, axial section, showing a lumbar disc herniation compressing the right L5 spinal nerve root.

**Figure 3:**
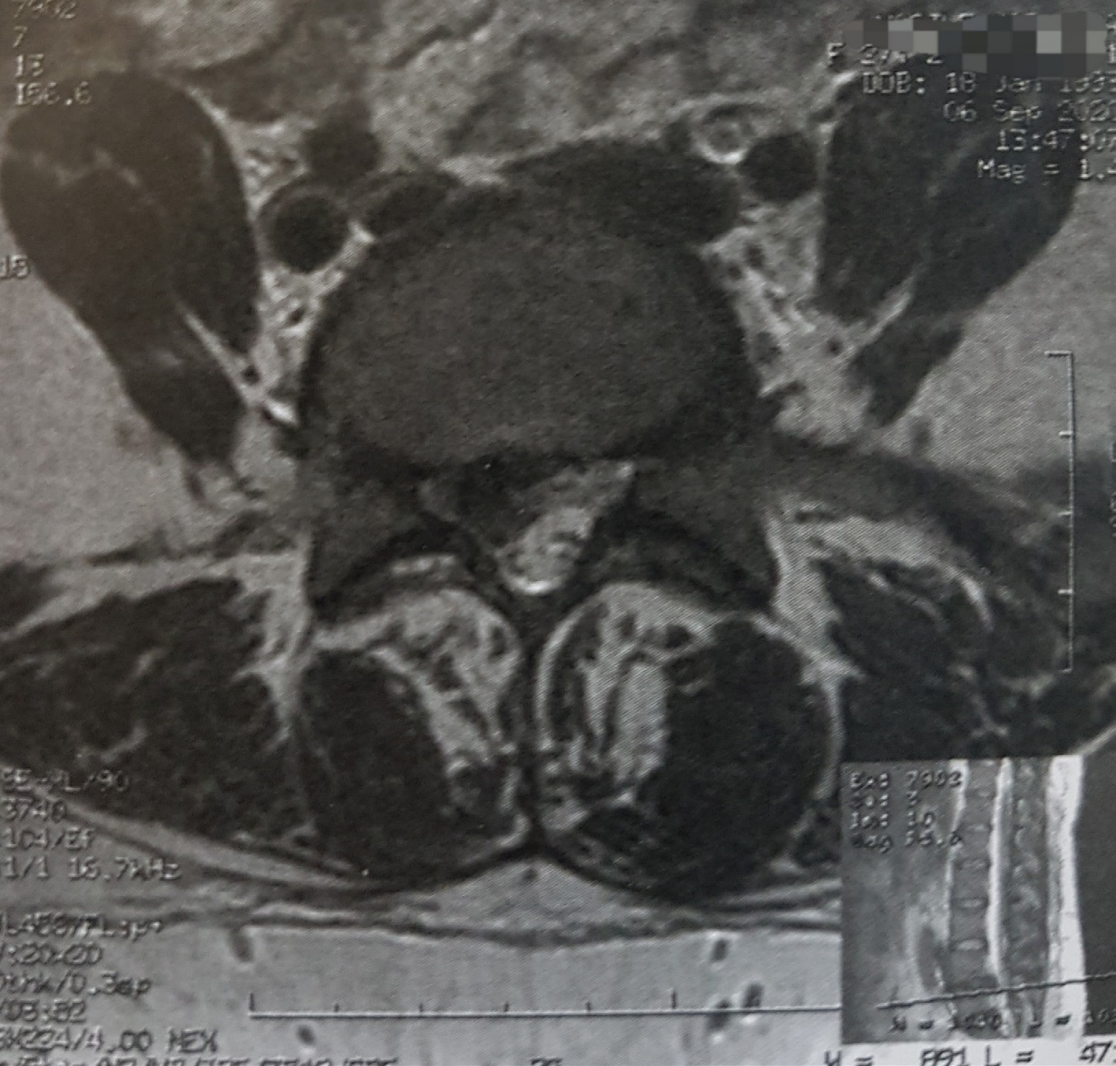
T2-weighted MRI scan, axial section, showing a caudally-migrated L4/L5 disc fragment.

A 27-year-old multiparous woman was referred to our department one month postpartum, with a 4-month history of LBP irradiating to her right lower limb. The patient had an uncomplicated vaginal birth and reported progressive worsening of the symptoms in the following month, starting the day after delivery. Neurological examination showed radicular pain, hypoesthesia and paresthesia in the right L5 and S1 nerve territory, weakness of the right extensor halluces longus (great toe dorsiflexion) and reduced deep tendon reflex of the Achilles tendon. Magnetic resonance imaging (MRI) examination showed a large right L4/L5 disc hernia (Figures 1, 2) with a caudally-migrated disc fragment (Figure3). The patient underwent L4/L5 microdiscectomy, with an immediate improvement of the symptoms postoperatively and no neurological sequelae.

## Discussion

Although the most common spinal pathology during pregnancy is symptomatic lumbar disc herniation [11], this disorder is extremely rare compared to pregnancy-related LBP, being estimated to appear 1 in 10,000 patients [2]. Because of the increase in the mean age of women becoming pregnant noted in recent years, an increase in the incidence of lumbar disc hernia in pregnant women is to be expected. It has been reported that the proportion of children born by women older than 35 years raised from 4% to 21% between 1990 and 2015 [12]. Nine out of 10 cases reviewed were patients older than 30 years, 4 were over 35 years old. It was postulated that hormonal changes, in particular serum relaxin increase, may lead to LBP, as well as pain in the sacral and pelvic region [13]. However, the most common symptom of lumbar disc herniation is radicular pain, which may or may not be associated with LBP [11]. Out of the 10 cases reviewed in this article, 2 presented only with LBP, while the other 8 presented with either urinary incontinence, radicular pain, diminished sensation in the sensory distribution of one spinal nerve, or weakness in the muscles innervated by the motor root of a lumbar spinal nerve. Cauda equina syndrome, a criterion for emergency neurosurgical intervention, consists of radiating pain or numbness and bilateral muscle weakness involving both lower extremities, bladder, or bowel dysfunction [11]. It has been reported in the literature that no more than 15% of the patients suffering from lumbar disc hernias develop severe neurologic deficits [3]. In this study, we reviewed a case of CES at presentation; the patient underwent laminectomy immediately following the cesarean section (CS) and had no neurological sequelae [7]. MRI during pregnancy has gained a more widely accepted acceptance, but the exact risk for the fetus has yet to be clearly established, and research still needs to be undertaken in this area [14]. All cases reviewed in this article underwent an MRI scan, which revealed lumbar disc hernia at either the L5/S1 level (6 cases) or L4/L5 level (4 cases). Regarding therapeutic management, it has been stated that most patients suffering from lumbar disc hernia can heal without surgery [4]. Six out of 10 patients underwent conservative treatment and had no residual pain or neurological deficit after delivery. One patient presented at 33 weeks with radicular pain and developed motor weakness during the following week. The patient delivered by cesarean section, underwent discectomy immediately after, and was discharged without any neurological deficit [9]. Another patient received an epidural steroid injection, and her symptoms improved, with no neurological sequelae [10].

Regarding the delivery method, the literature data are still controversial about lumbar disc hernias associated with pregnancy, reporting anecdotical cases, or very small case series. The decision to treat conservatively (“wait and see”) or surgically seems to be influenced by the cooperation between the obstetrician and neurosurgeon, based on MRI data.

Postpartum evolution was unpredictable (cesarean section offered to pregnant women with lumbar disc hernia followed by remission of the symptoms and no neurological deficit [2, 9]; antepartum epidural steroid injection, vaginal delivery and no neurological deficit in the postpartum period [10]; cesarean section for failed labor induction at term, followed by immediate microdiscectomy due to CES [8]; operative vaginal delivery using vacuum extraction, followed at 4 weeks postpartum by fecal and urinary incontinence, not improved by discectomy [6]; spontaneous vaginal deliveries followed by CES).

The analysis of these cases suggests labor could increase the risks of neurological symptoms worsening or CES development in the postpartum period and that CS might be the safer option for pregnant women with symptomatic lumbar disc hernia.

## Conclusion

The best delivery method for pregnant women suffering from lumbar disc hernia is not clearly defined. The paucity of cases and limited experience are the explanation. The best decision and results seem to be related to the cooperation between the obstetricians and neurosurgeons and MRI data. Based on limited data, CS seems to be preferred compared to vaginal delivery in terms of avoiding symptom worsening and progression to CES, but further research is required.

## Conflict of Interest

The authors declare that there is no conflict of interest.
